# Supplementary material for: New Insights on the Nutrition Status and Antioxidant Capacity in Multiple Sclerosis Patients
Source: Nutrients. 2019 Feb 18;11(2):427. doi: 10.3390/nu11020427 (PMC6413226; doi:10.3390/nu11020427)
Supplement: Supplementary file 1 [file nutrients-11-00427-s001.pdf]

Table S1. Comparison of routine blood test values between different MS treatments.

Variables	Interferon Beta	Fingolimod	Other Medication	MS Untreated	*p* *
Iron (µg/dL)	74.3±26.5	62.8±20.8	49.3±56.0	54.9±25.2	0.167
Ferritin (ng/mL)	92.9±81.5	46.6±20.2	98.4±72.8	49.1±57.2	0.113
Vitamin B12 (pg/mL)	416.1±175.6	403.6±157.3	496.6±196.9	488.7±167.6	0.225
Magnesium (mg %)	2.1±0.1	2.1±0.1	2.1±0.1	2.2±0.1	0.502
Folic acid (ng/mL)	9.5±5.2	11.7±4.7	7.4±5.0	12.7±4.7	0.064
Triglycerides (mg/dL)	108.2±57.3	123.4±57.1	90.7±35.4	121.8±106.6	0.664
Total Cholesterol (mg/dL)	172.8±26.8	175.8±27.3	184.3±30.1	183.8±47.0	0.775
HDL (mg/dL)	46.5±8.4	50.8±20.4	53.8±13.4	46.4±12.7	0.985
LDL (mg/dL)	103.3±22.8	102.7±17.5	116.0±26.7	122.1±40.2	0.449
Albumin (g %)	4.1±0.4	4.1±0.3	3.9±0.5	4.0±0.7	0.941

Kruskal–Wallis non-parametric test was used for analysis. Abbreviations: multiple sclerosis (MS), low-density lipoprotein (LDL), high-density lipoprotein (HDL). Values of routine blood tests are reported as mean ± SD. Of the 63 MS patients, there were 24 patients treated with Interferon beta (15 Rebif, 8 Avonex, and 1 Betaferon), 10 treated with Fingolimod (Gilenya), 14 treated with other medications (3 Tysabri, 3 Dimethyl Fumarate, 3 Glatiramer acetate, 3 Aubagio and 2 ocrelizumab) and 15 patients untreated at enrolment time.

Table S2. Comparison of fatty acid profiles from membranes of RBC between different MS treatment groups.

Fatty acid profiles	Interferon Beta	Fingolimod	Other Medications	MS Non Treated	p
C14_0	0.29±0.17	0.31±0.11	0.25±0.10	0.23±0.16	0.281
C16_0	21.9±1.2	21.9±1.2	21.5±0.7	21.3±1.0	0.357
C16_1n7t	0.05±0.03	0.07±0.02	0.06±0.04	0.05±0.02	0.058
C16_1n7	0.27±0.20	0.32±0.31	0.26±0.13	0.25±0.26	0.648
C18_0	16.4±2.0	16.2±2.3	16.5±1.6	17.5±1.8	0.291
C18_1t	0.25±0.09	0.30±0.12	0.35±0.19	0.29±0.14	0.307
C18_1n9	14.9±1.4	15.4±1.5	15.1±1.5	14.4±1.6	0.552
C18_2n6tt	0.04±0.04	0.03±0.03	0.04±0.05	0.03±0.05	0.700
C18_2n6ct	0.03±0.02	0.03±0.03	0.02±0.01	0.02±0.02	0.377
C18_2n6tc	0.09±0.03	0.10±0.03	0.11±0.05	0.09±0.03	0.570
C18_2n6	13.3±3.3	14.3±3.9	13.1±2.8	11.8±2.5	0.180
C20_0	0.16±0.05	0.18±0.07	0.16±0.04	0.16±0.06	0.883
C18_3n6	0.09±0.06	0.12±0.06	0.10±0.05	0.09±0.06	0.226
C20_1n9	0.24±0.06	0.25±0.06	0.26±0.05	0.26±0.08	0.709
C18_3n3	0.18±0.14	0.21±0.11	0.15±0.09	0.12±0.07	0.258
C20_2n6	0.30±0.07	0.28±0.05	0.31±0.04	0.30±0.06	0.551
C22_0	0.30±0.13	0.35±0.17	0.27±0.12	0.32±0.13	0.572
C20_3n6	1.72±0.29	1.57±0.16	1.78±0.35	1.60±0.32	0.271
C20_4n6	16.3±2.3	16.2±2.3	16.6±2.0	17.1±1.4	0.798
C24_0	0.92±0.35	0.84±0.37	0.92±0.34	1.04±0.39	0.551
C20_5n3	0.48±0.44	0.52±0.47	0.52±0.34	0.52±0.27	0.640
C24_1n9	0.93±0.40	0.86±0.31	0.98±0.38	1.12±0.48	0.660
C22_4n6	3.83±1.34	3.39±1.26	3.61±0.89	3.54±0.92	0.874
C22_5n6	1.06±0.38	0.79±0.26	1.07±0.23	1.05±0.32	0.135

C22_5n3	1.88±0.40	1.86±0.43	1.89±0.48	2.23±0.65	0.354
C22_6n3	4.07±1.39	3.48±0.92	4.13±1.80	4.63±1.31	0.278
Omega-3 Index	4.34±1.70	3.64±0.84	4.38±1.86	4.95±1.40	0.303

Analysis of fatty acids was performed via gas chromatography. Values are reported as mean±SD. Kruskal–Wallis non-parametric test was used for analysis. Abbreviations: Multiple sclerosis (MS); Omega-3 Index was calculated as sum of Eicosapentaenoic acid and Docosahexaenoic acid. Of the 63 MS patients, there were 24 patients treated with Interferon beta (15 Rebif, 8 Avonex, and 1 Betaferon), 10 treated with fingolimod (Gilenya), 14 treated with other medications (3 Tysabri, 3 Dimethyl Fumarate, 3 Glatiramer acetate, 3 Aubagio and 2 ocrelizumab) and 15 patients untreated at enrolment time.
